# A case of adenomyosis with leiomyoma that was effectively treated with relugolix and kamishoyosan add-on therapy

**DOI:** 10.1186/s12905-021-01442-x

**Published:** 2021-08-19

**Authors:** Yukifumi Sasamori, Kohei Takehara, Tsuyoshi Terashima, Takako Onodera, Keita Yatsuki, Ippei Nakagawa, Yuko Takahashi, Haruka Nishida, Takayuki Ichinose, Haruko Hiraike, Kazunori Nagasaka

**Affiliations:** Department of Obstetrics and Gynecology, Teikyo University School of Medicine, Tokyo , Japan

**Keywords:** Leiomyoma, Adenomyosis, GnRH antagonist, Relugolix, Kamishoyosan

## Abstract

**Background:**

Recently, relugolix, an oral gonadotropin-releasing hormone receptor antagonist, has been considered an effective therapy for leiomyoma based on a phase 3 study in Japanese women. Leiomyoma combined with severe adenomyosis occasionally occurs in perimenopausal women; however, little information on the effectiveness of relugolix against severe adenomyosis exists.

**Case presentation:**

A 49-year-old woman was referred to our hospital with acute lower abdominal pain and abnormal uterine bleeding. Magnetic resonance imaging revealed multiple leiomyomas with diffuse adenomyosis. Left hydrosalpinx was also observed. The patient refused surgical treatment and preferred oral relugolix. Since she experienced a hot flush and headache induced by relugolix, a traditional Japanese Kampo, kamishoyosan, was added to improve the side effects of relugolix. The patient was asymptomatic at the time of this report and experienced a significant shrinkage in uterine volume. Ultimately, she avoided hysterectomy as desired.

**Conclusions:**

To our knowledge, this is the first report of co-occurring adenomyosis and leiomyoma, which was effectively treated with relugolix. Although the management of adverse side effects, including hot flush and headache by relugolix, has recently attracted attention and controversy, relugolix add-on therapy with kamishoyosan may help treat menopausal symptoms.

## Background

Uterine leiomyomas are common in Japanese women, although the incidence has not been well investigated [1]. In the United States and Europe, the incidence may be higher than in Japan, and, possibly, more than 70% of women are affected by leiomyoma by the age of 50 years in the world [2, 3]. Symptomatic leiomyoma often presents with dysmenorrhea, severe anemia, and prolonged abnormal uterine bleeding (AUB) [4]. Some cases are frequently accompanied by adenomyosis in susceptible age groups [5]. Adenomyosis is a chronic inflammatory disease that is associated with dysmenorrhea and severe pelvic pain [6]. Generally, adenomyosis is noted after 40 years of age [7]. Recently, the PALM-COEIN (polyp, adenomyosis, leiomyoma, malignancy, hyperplasia, coagulopathy, ovulatory dysfunction, endometrial, iatrogenic, and not-yet-classified) classification system for AUB was approved by the International Federation of Gynecology and Obstetrics [8]. Uterine leiomyoma and adenomyosis are benign conditions, however, are considered to be leading causes of AUB and generally, regress after menopause [8]. Cases of adenomyosis with leiomyoma are often encountered in perimenopausal women, and some coexist with endometrial hyperplasia and endometrial polyps [7, 9]. However, the pathophysiology underlying the comorbidities of these diseases remains unclear. Recent investigations have reported certain genetic risk factors for estrogen-dependent endometrial cancer [10]. Treatment for adenomyosis and leiomyoma in perimenopausal women should be individualized considering the symptomatology. Almost half of asymptomatic cases, including unaware cases, would require sustained follow-up, measuring the size and location by ultrasound examination [4]. Masses are occasionally misdiagnosed, and in such cases, magnetic resonance imaging (MRI) provides additional information [11]. T2-weighted imaging is often helpful in differentiating between adenomyosis and leiomyoma. Typically, adenomyosis appears as an ill-demarcated low-signal-intensity lesion with uterine enlargement [11]. In contrast, well-circumscribed lesions with homogenous hypointensity can be found in most leiomyomas [12]. After total pelvic evaluations, most women approaching perimenopausal age often need to decide between surgical treatment or waiting for menopause. Hysterectomy is one surgical option for the management of leiomyomas [13]. The types of hysterectomy vary based on surgeon training and approach (abdominal, laparoscopic, robot-assisted, or vaginal). For an enlarged uterine mass, total abdominal hysterectomy is occasionally chosen as the standard treatment. Recently, laparoscopic techniques have been successfully adapted to the general sized uteri with fewer complications, leading to their increased use [14]. Myomectomy is a surgical option that aims to preserve the uterus. Some procedures are performed laparoscopically with minimal invasiveness. However, many women desire non-surgical options. For these cases, hypoestrogenic therapy with gonadotropin-releasing hormone (GnRH) analogs has been widely used [15]. Recently, women with leiomyoma were successfully treated for AUB and pain with relugolix in Japan [16, 17]. Relugolix is an oral non-peptide GnRH-receptor antagonist without the flare-up symptoms commonly associated with GnRH analogs [16, 17]. In Japan, oral relugolix was approved for improving various symptoms, including menorrhagia, lower abdominal pain, back pain, and anemia, based on uterine leiomyoma and was covered by national insurance in March 2019 [18]. Few cases in the literature indicate that oral relugolix is now widely used for the treatment of leiomyoma. However, our search revealed no reports on the use of relugolix for treating adenomyosis, although a few studies have reported using GnRH antagonists because of their effectiveness in treating this condition [19, 20]. Herein, we describe the case of a woman with leiomyoma, who also was found to have adenomyosis. We, incidentally found elevated C-reactive protein levels and white blood cell count in response to inflammation in the uterus. To the best of our knowledge, this is the first report of leiomyoma with adenomyosis that was treated with relugolix. Furthermore, the side effects of relugolix induced by hypoestrogenic conditions were effectively relieved with kamishoyosan, a Japanese traditional Kampo medicine, as an add-on therapy with relugolix.

## Case presentation

**Fig. 1:**
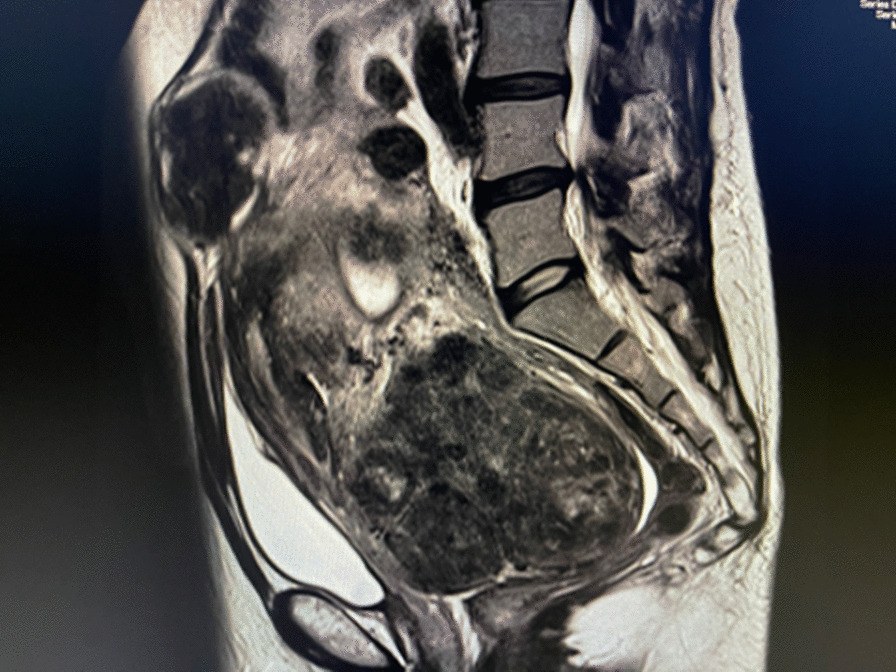
Magnetic resonance images showing an enlarged bicornuate bicollis uterus with diffuse adenomyosis. MRI showing bicornuate bicollis uterus with multiple leiomyomas, diffused adenomyosis, and multiple hematomas in the uterus. Size of the uterus, including fibroid and adenomyosis, measured 22 cm in length and nearly 10 cm in thickness

A 49-year-old woman, gravida 0, was referred to our hospital with severe acute lower abdominal pain and AUB. Her general physical examination was normal apart from pelvic pain and a distended abdomen due to an enlarged uterus. She had never undergone a medical assessment. Therefore, we first performed sampling cytology of the cervix and endometrium. Ultrasonography showed multiple leiomyomas in the bicornuate bicollis uterus with hydrosalpinx in the left tube. The work-up revealed anemia, a high level of D-dimer, and inflammation. The patient’s laboratory results are summarized in Table 1. A high level of serum CA-125 was also observed. No apparent deep vein thrombosis was observed in the legs. She refused hospitalization for further examinations; thus, we allowed her to rest at home with oral prophylactic antibiotic administration. MRI revealed a bicornuate bicollis uterus with multiple leiomyomas from the cervix to the fundus, which were up to 11 cm in size. Additionally, we detected adenomyosis, multiple hematomas in the uterus, and hydrosalpinx in the left tube, possibly induced by endometriosis (Fig. 1). To summarize, severe benign conditions were observed; however, no malignant lesions were detected on gynecologic examination.

**Table 1:** Laboratory results and serologies at the first medical examination

Parameter	
WBC	9.0 × 10^3^/µL
Hb	8.1 g/dL
Plt	3.2 × 10^4^/µL
TP	7.0 g/dL
Alb	3.8 g/dL
LDH	172 U/L
BUN	9.3 mg/dL
Cre	0.65 mg/dL
Na	138 mEq/L
K	3.9 mEq/L
Cl	103 mEq/L
AST	24 U/L
ALT	19 U/L
CRP	11.45 mg/dL
PT%	78%
PT-INR	1.12
APTT	32.4 s
D-dimer	2.4 µg/mL
CEA	1.7 ng/mL
CA19-9	9.9 U/mL
CA125	111.3 U/mL

Alb, albumin; ALT, alanine aminotransferase; APTT, alanine aminotransferase; AST, aspartate aminotransferase; BUN, blood urea nitrogen; CEA, carcinoembryonic antigen; Cre, creatinine; CRP, C-reactive protein; Hb, hemoglobin; LDH, lactate dehydrogenase; Plt, platelet; PT%, ; PT-INR, prothrombin time-international normalized ratio; TP, ; WBC, white blood cell

**Fig. 2:**
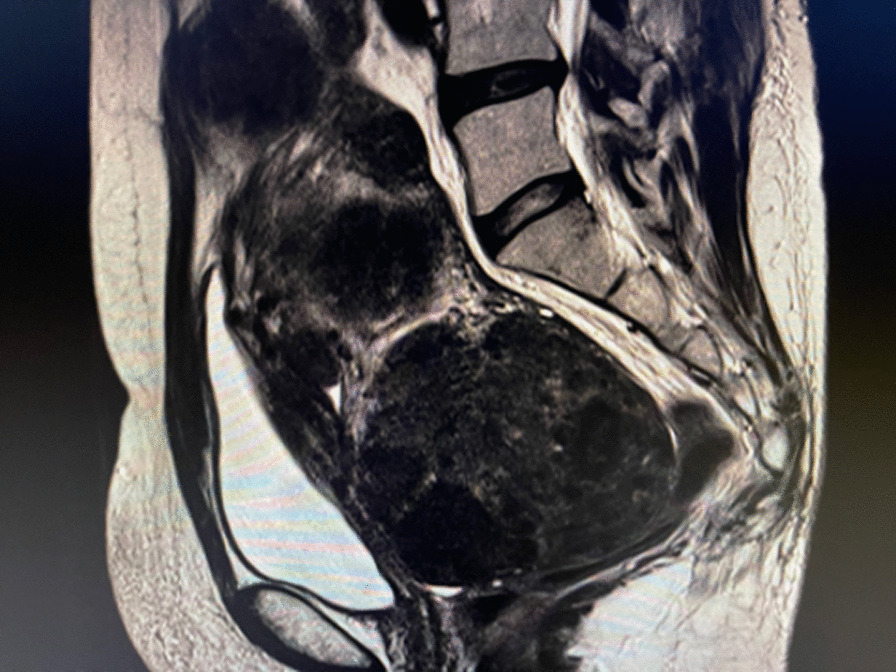
Magnetic resonance images showing a significant reduction of the uterus after 6 months of oral relugolix (40 mg/day). After relugolix therapy for 6 months, diffused adenomyosis mostly disappeared compared to the baseline. It is noteworthy that the size of the uterus, including fibroid and adenomyosis, shrunken to 15 cm in length and 8 cm in thickness

Based on these examinations and because her symptoms and laboratory data were improving compared to her first hospital visit, except for the continuous AUB with pelvic pain, we presented her with the treatment options (surgical or non-surgical) to cure her severe symptoms induced by leiomyoma and adenomyosis. Because her enlarged uterus had grown up to the xiphisternum, we presented hysterectomy as the first option. However, she refused and opted for non-surgical treatment. We prescribed oral relugolix (40 mg/day) as maintenance therapy. We assessed her condition within a month during the therapy and confirmed that her symptoms induced by the leiomyoma and adenomyosis were relieved, and she remained amenorrheic. Two months after the initial relugolix administration, she suffered from a hot flush with a slight headache, which was considered to be induced by relugolix. We discussed with the patient about discontinuing the relugolix therapy and recommended surgical treatment. After 6 months of treatment with relugolix, we evaluated her hip and spine bone mineral density measured by dual-energy X-ray absorptiometry, and the results were normal. We found that the uterine volume had significantly decreased on MRI, and the adenomyosis had mostly disappeared compared to the baseline image (Fig. 2). We continued follow-up with gynecologic examinations via ultrasonography, and after another 6 months, she experienced AUB recurrence; however, the bleeding was less compared with the baseline. The patient still desired continued alleviation of symptoms by relugolix therapy with the addition of kamishoyosan at a dose of 7.5 g/day before each meal. Undesirable reported adverse events, such as itchiness, rash, nausea, constipation and gastric discomfort, were not found. Nothing was quantified in a QOL analysis, however, kamishoyosan clearly improved the adverse vasomotor effects induced by relugolix. Therefore, we repeated the workup, including sampling cytology from the uterus, and retreated her with oral relugolix and kamishoyosan for 6 months, which is the duration confirmed in clinical studies. The patient is currently asymptomatic.

## Discussion and conclusions

Uterine leiomyoma and adenomyosis are considered estrogen-dependent and, not surprisingly, these benign diseases have overlapping symptomatology and reproductive consequences [21–23]. Both lesions can grow to notably large sizes; in such cases, surgery is strongly recommended to patients. Recent clinical phase 3 trials reported that relugolix combination therapy with 1 mg of estradiol and 0.5 mg of norethindrone acetate significantly improved abnormal bleeding with minor adverse vasomotor events, including hot flushes and headaches [23, 23]. In our case, we did not prescribe the relugolix combination therapy, but used a traditional Japanese Kampo medicine, kamishoyosan, to ameliorate the adverse vasomotor effect induced by continuous relugolix administration for 2 months. Fortunately, the patient’s bone mineral density was unaffected by the prolonged administration of relugolix for more than 6 months; future investigation is required to clarify this effect. Kamishoyosan contains herbal medicines, including bupleurum root, ginger, rhizomes of Atractylodes lancea, and Moutan bark, and is indicated for menopausal symptoms, with side effects, such as hot flushes, shoulder stiffness, and neuropsychiatric symptoms, including depression and irritability [24]. To the best of our knowledge, it is the first report to show the effectiveness of relugolix against both leiomyomas and adenomyosis with severe lower abdominal pain and continuous AUB. Relugolix significantly reduced the size of leiomyoma and diminished adenomyosis (Fig. 2). The adverse side effects of relugolix were also relieved by add-on therapy with kamishoyosan. In conclusion, relugolix, an oral GnRH antagonist, should improve symptoms in perimenopausal women who experience pelvic discomfort and could serve as an option to the standard therapy for leiomyoma with adenomyosis.

## Data Availability

The datasets used and/or analysed during the current study available from the corresponding author on reasonable request.

## Abbreviations

AUB: Abnormal uterine bleeding
GnRH: Gonadotropin-releasing hormone
MRI: Magnetic resonance imaging
